# Bax regulates c-Myc-induced mammary tumour apoptosis but not proliferation in MMTV-c-*myc* transgenic mice

**DOI:** 10.1038/sj.bjc.6602137

**Published:** 2004-08-31

**Authors:** M H Jamerson, M D Johnson, S J Korsmeyer, P A Furth, R B Dickson

**Affiliations:** 1 Department of Oncology and Lombardi Comprehensive Cancer Center, Georgetown University Medical Center, 3970 Reservoir Road, NW, Washington, DC 20057, USA; 2 Department of Cancer Immunology and AIDS and Howard Hughes Medical Institute, Dana Farber Cancer Institute, Harvard University, 44 Binney Street, Boston, MA 02115, USA

**Keywords:** Bax, c-Myc, transgenic mice, mammary gland, apoptosis, tumorigenesis

## Abstract

The expression of the proto-oncogene c-*myc* is frequently deregulated, via multiple mechanisms, in human breast cancers. Deregulated expression of c-*myc* contributes to mammary epithelial cell transformation and is causally involved in mammary tumorigenesis in MMTV-c-*myc* transgenic mice. c-Myc is known to promote cellular proliferation, apoptosis, genomic instability and tumorigenesis in several distinct tissues, both *in vivo* and *in vitro*. Expression of the proapoptotic regulatory gene *bax* is reduced or absent in human breast cancers, and c-Myc has been shown to regulate the expression of Bax, as well as cooperate with Bax in controlling apoptosis in a fibroblast model. Additionally, loss of *bax* reduces c-Myc-induced apoptosis in lymphoid cells and increases c-Myc-mediated lymphomagenesis *in vivo*. In order to assess whether loss of *bax* could influence c-Myc-induced apoptosis and tumorigenesis in the mammary gland *in vivo*, we generated MMTV-c-*myc* transgenic mice in which neither, one, or both wild-type alleles of *bax* were eliminated. Haploid loss of *bax* in MMTV-c-*myc* transgenic mice resulted in significantly reduced mammary tumour apoptosis. As anticipated for an apoptosis-regulatory gene, loss of the wild-type *bax* alleles did not significantly alter cellular proliferation in either mammary adenocarcinomas or dysplastic mammary tissues. However, in contrast to c-Myc-mediated lymphomagenesis, loss of one or both alleles of *bax* in MMTV-c-*myc* transgenic mice did not significantly enhance mammary tumorigenesis, despite evidence that haploid loss of *bax* might modestly increase mammary tumour multiplicity. Our results demonstrate that Bax contributes significantly to c-Myc-induced apoptosis in mammary tumours. In addition, they suggest that in contrast to c-Myc-induced lymphomagenesis, mammary tumorigenesis induced by deregulated c-*myc* expression requires some amount of Bax expression.

The proto-oncogene c-*myc* was first identified as the mammalian homologue of the viral transforming oncogene, v-*myc*, responsible for avian myelocytomatosis (Vennstrom *et al*, 1982). c-Myc is a DNA-binding, nuclear transcription factor involved in the regulation of cell cycle progression, programmed cell death, cellular metabolism, and differentiation (Evan and Littlewood, 1993; Harrington *et al*, 1994; Packham and Cleveland, 1995). In 1994, the Dual Signal model proposed that induction of apoptosis, a potent mechanism for the suppression of tumorigenesis, was an obligate function of deregulated c-*myc* expression; however, more recent experimentation suggests that c-Myc may sensitise cells to apoptosis as a result of alterations in mitochondrial membrane permeability and movement of holocytochrome *c* into the cytoplasm from its typical position as a constituent of the electron transport system (Juin *et al*, 1999; Prendergast, 1999). Experiments examining the cooperation of c-Myc and knockouts of p19^ARF^ and/or p53 in mouse embryo fibroblast models have suggested that suppression of c-Myc-induced apoptosis may facilitate malignant transformation and tumorigenesis (Zindy *et al*, 1998). c-Myc may increase genomic instability and enhance tumorigenesis without an absolute requirement for continued aberrant c-*myc* expression once additional transforming genetic lesions have been generated and fixed in the genome (Felsher and Bishop, 1999a, 1999b). Deregulated or aberrant expression of c-*myc*, via mechanisms including translocation, proviral insertion, locus amplification, point mutation, direct transcriptional and translational effects, or post-translational modifications, is a signature of several different human tumour types and c-*myc* can induce tumorigenesis under conditions where programmed cell death is abrogated (Evan *et al*, 1992; Santoni-Rugiu *et al*, 1998; Dang, 1999). The relevance of aberrant c-*myc* expression to the pathogenesis of breast cancer is confirmed by the finding that the c-*myc* locus is rearranged in roughly 5%, amplified in 16%, and overexpressed in approximately 70% of human breast tumours (Nass and Dickson, 1997; Deming *et al*, 2000).

The role of c-*myc* expression in normal mammary gland development and function as well as mammary tumorigenesis is a burgeoning field of inquiry. Several *in vitro* studies have demonstrated a contributory role for c-Myc in transformation of both human and murine mammary epithelial cells (MECs) (Leder *et al*, 1986; Telang *et al*, 1990; Valverius *et al*, 1990). Examination of normal murine mammary development has indicated that c-Myc is expressed during pregnancy-associated proliferation and postlactational involution associated MEC apoptosis (Strange *et al*, 1992). To further evaluate the role of c-Myc in mammary development, function, and transformation, a transgenic mouse was generated expressing the murine c-*myc* gene under the control of the mouse mammary tumour virus long terminal repeat (MMTV-LTR) promotional elements (Stewart *et al*, 1984). MMTV-c-*myc* transgenic mice develop mammary adenocarcinomas in both the virgin state (∼50% incidence following a 7–14 month latency) and the multiparous state (∼100% incidence with two or more pregnancies); however, the extended mammary tumour latencies and low mammary tumour multiplicities suggest that c-*myc* is contributory to but insufficient for mammary tumorigenesis in the mouse (Stewart *et al*, 1984; Leder *et al*, 1986). The conditional expression of c-*myc* in the mammary glands of mice using an MMTV-LTR-driven tetracycline-responsive transgenic system has provided evidence for cooperative, transforming genetic alterations that may result from c-Myc-induced genomic instability (D'Cruz *et al*, 2001). Furthermore, the use of spectral karyotyping (SKY) and comparative genomic hybridisation (CGH) to demonstrate that MMTV-c-*myc*-induced mammary tumours display distinct, repeatable patterns of chromosomal alterations suggests that c-Myc may exert a dominant genomic mutator effect and that specific genetic lesions may cooperate in MEC transformation (reflecting the multistage nature of human tumorigenesis) (Weaver *et al*, 1999).

TGF*α*, a soluble growth factor of the epidermal growth factor family of ligands, is a potent survival and growth factor for human and murine MECs both *in vivo* and *in vitro*, and when overexpressed in the mammary glands of transgenic mice, induces mammary alveolar hyperplasias and occasional mammary adenocarcinomas (Bates *et al*, 1990; Jhappan *et al*, 1990; Matsui *et al*, 1990; Sandgren *et al*, 1990; Snedeker *et al*, 1991; Amundadottir *et al*, 1995). The prosurvival molecule Bcl-2 has been shown to be expressed in the normal human and murine mammary epithelium, to impede mammary gland involution when expressed as an exogenous transgene, and to suppress c-*myc*-induced apoptosis and cooperate with c-*myc* expression in inducing B-cell malignancies in another transgenic model (Bargou *et al*, 1995; Schorr *et al*, 1999a, 1999b; Feuerhake *et al*, 2000; Eischen *et al*, 2001b). The importance of suppression of apoptosis in c-*myc*-induced murine mammary tumorigenesis has been suggested by three independently generated transgenic mouse models: MMTV-c-*myc*/MT-*tgfα*, WAP-c-*myc*/WAP-*tgfα*, and MMTV-c-*myc*/WAP-*bcl2* (Amundadottir *et al*, 1995; Sandgren *et al*, 1995; Jäger *et al*, 1997). Data from these three mammary bitransgenic studies strongly suggest that mammary tumorigenesis is significantly enhanced when deregulated c-*myc* expression, responsible for driving both proliferation and apoptosis, is coupled with alterations that block c-*myc*-mediated apoptotic pathways.

Bax, a proapoptotic member of the Bcl-2 family of proteins, was first discovered in a screen of proteins that exhibited binding interactions with Bcl-2 (Oltvai *et al*, 1993). Bax is likely to have pore-forming activity in the mitochondrial membranes, subject to control or prevention by association with specific antiapoptotic molecules (especially Bcl-2 and Bcl-x_L_), related to its ability to bind to BH-3 domain-only containing Bcl-2 family member proteins, and induce the release of mitochondrial cytochrome *c* (Antonsson *et al*, 1997; Jurgensmeier *et al*, 1998; Desagher *et al*, 1999; Murphy *et al*, 1999; Antonsson *et al*, 2000; Nouraini *et al*, 2000; Wei *et al*, 2001). Bax is weakly expressed or absent in several breast cancer cell lines and transfection of *bax* into these lines results in increased apoptotic sensitivity and diminished tumour proliferation in athymic mice (Bargou *et al*, 1995, 1996; Sakakura *et al*, 1996). Bax is expressed in the epithelium of the normal breast and its expression is highest during postlactational mammary gland involution; furthermore, Bax expression is significantly reduced or absent in invasive ductal breast carcinomas (Krajewski *et al*, 1994; Bargou *et al*, 1995; Li *et al*, 1996; Feuerhake *et al*, 2000; Shilkaitis *et al*, 2000). Significant reductions in Bax expression were found in 34% of primary breast tumours in women with metastatic disease, Bax expression was inversely correlated with overall survival, treatment response, and metastasis in these patients, and Bax expression was found to be predictive of tumour response to chemotherapy independent of other predictive variables (Krajewski *et al*, 1995; Kapranos *et al*, 1997; Sjöström *et al*, 1998).

The mechanisms by which c-Myc induces apoptosis and the manner in which this apoptosis contributes to tumour suppression are largely unknown and currently being explored. Recently, *bax* was determined to be transcriptionally regulated by c-Myc in a variety of human cell lines (including the SkBr3 human breast cancer cell line) and found to be critical for the induction of apoptosis by aberrant c-Myc expression in a mouse embryo fibroblast model system (Mitchell *et al*, 2000). Two other studies indicate that c-Myc, at least in embryo fibroblast systems, activates a proapoptotic function in Bax and induces an apoptotic program that requires Bax (or a BH3 domain peptide) to be present in the mitochondrial membrane (Soucie *et al*, 2001; Juin *et al*, 2002). In addition, *bax*-deficient primary pre-*β* cells have been shown to be resistant to proapoptotic effects of c-Myc. Furthermore, in a transgenic mouse model, loss of one or both *bax* allele(s) significantly accelerate c-Myc-dependent lyphomagenesis in a *bax* gene dosage-dependent manner (Eischen *et al*, 2001a). The partial or total loss of *bax* in knockout mice provides evidence that the presence of Bax is unlikely to be required for mammary gland development and secretory differentiation (a very small percentage of *bax*-nullizygous mice did evidence postpartum lactational incompetency); however, loss of *bax* did reduce MEC apoptosis during postlactational involution (Knudson *et al*, 1995; Schorr *et al*, 1999a, 1999b). Loss of *bax* (reflecting the *in vivo* situation of human breast cancer patients) may disrupt c-Myc-induced apoptotic programs in mammary epithelial cells and has the potential to diminish the tumour suppressive activity of c-Myc-induced apoptosis. In this study, we have generated a combinatorial, mammary-relevant transgenic model, *bax*-knockout/MMTV-c-*myc* transgenic, to examine the influence of allelic *bax* loss on c-Myc-induced apoptosis and tumorigenesis *in vivo*.

## MATERIALS AND METHODS

### Transgenic and knockout mice

All animal experiments were conducted in accordance with US and UK CCR guidelines (Workman *et al*, 1998) and in accordance with our institutionally approved protocol. MMTV-c-*myc* transgenic mice (FVB inbred genetic background) were obtained from Charles River Laboratories, bred under a license from DuPont Medical Products, and housed as previously described (Amundadottir *et al*, 1995). The MMTV-c-*myc* transgenic mice contain a mouse mammary tumour virus long terminal repeat promoter element driving the expression of a murine c-*myc* gene (Stewart *et al*, 1984). Mice hemizygous for *bax* (C57BL/6 inbred genetic background) were obtained from SJ Korsmeyer via PA Furth at Georgetown University (Knudson *et al*, 1995). P generation *myc* mice were bred to P generation *bax*-hemizygous mice and subsequently, their F_1_ generation offspring were backcrossed with P generation *bax*-hemizygous mice, resulting in F_2_ generation offspring in which the *myc* transgene was found in the context of no, one, or two intact wild-type *bax* alleles. All data reported herein were generated using F_2_ generation study mice on the mixed genetic background (C57BL/6 × FVB; 3 : 1). Parous study mice, 10 weeks old, were housed with males and repetitively bred until euthanisation; all surviving pups were weaned at day 20 postpartum. Female parous study mice were examined bi-weekly for tumours and morbidity and euthanased if they showed signs of ill health using approved methods (Workman *et al*, 1998).

### Genotyping

Overnight digestion of mouse tail biopsy samples (Workman *et al*, 1998) in STE buffer (0.1 M NaCl, 0.05 M Tris pH 8.0, 1 mM EDTA, and 1% SDS) containing 5 mg ml^−1^ fungal proteinase K (Invitrogen, Carlsbad, CA, USA), followed by phenol/chloroform extraction and ethanol precipitation yielded genomic DNA subsequently used in genotyping of all mice utilised in this study. PCR-based genotyping was performed on a Stratagene Robocycler Gradient 40 machine using tail-derived genomic DNA, sequence-specific primers, and Platinum PCR Supermix (Invitrogen). MTVMyc5′ primer (5′-CCC AAG GCT TAA GTA AGT TTT TGG-3′) and MTVMyc3′ primer (5′-GGG CAT AAG CAC AGA TAA AAC ACT-3′) were used to identify MMTV-c-*myc* transgenic mice (1 min denaturation at 95°C, 1 min annealing at 52°C, and 75 s elongation at 72°C for a total of 42 cycles); transgene-positive animals were identified by resolution of a single ∼880 bp band on a 1.0% agarose gel. BPR2 primer (5′-GTT GAC CAG AGT GGC GTA GG-3′), MK1 primer (5′-GAG CTG ATC AGA ACC ATC ATG-3′), and NRP2 primer (5′-CCG CTT CCA TTG CTC AGC GG-3′) were used to determine the allelic status of *bax* (45 s denaturation at 94°C, 90 s annealing at 55°C, and 2 min elongation at 72°C for a total of 35 cycles); animals with *bax* in the wild-type configuration demonstrate a single ∼300 bp band, animals nullizygous for *bax* demonstrate a single ∼506 bp band, and animals hemizygous for *bax* demonstrate both bands on a 1.0% agarose gel.

### Mammary gland tumour collection, histopathology, and whole-mount preparation

Mammary gland tumours and tissues were freshly collected via routine dissection procedures and split for fixation, liquid N_2_ snap-freezing (for molecular analyses), and whole-mount preparation. Mammary gland tumours and tissues were fixed in 10% neutral-buffered formalin (EM Sciences, Gibbstown, NJ, USA) in phosphate-buffered saline (PBS), embedded in paraffin, and sectioned by microtomy to 5 *μ*M. Mammary tumour and tissue sections were stained using haematoxylin and eosin and were subjected to histopathological evaluation using light microscopy. Mammary gland tissues for whole-mount preparation, routinely taken from the inguinal glands unless otherwise tumour involved, were fixed in 75% ethanol/25% glacial acetic acid, stained in a 0.2% carmine alum (Sigma, St Louis, MO, USA)/0.5% aluminum potassium sulfate solution, dehydrated through an ethanol series, cleared in toluene, mounted using Permount (Fisher, Fair Lawn, NJ, USA), and examined using dissecting stereomicroscopy.

### Western blot analyses

Mammary gland tumour and tissue samples that were harvested from the study mice were immediately snap-frozen in liquid N_2_, stored at −80°C, and later thawed in a RIPA homogenisation solution (× 1 PBS containing 1% NP-40, 0.5% sodium-deoxycholate, 0.1% SDS, and 10 *μ*g ml^−1^ PMSF). Briefly, samples were weighed, ground into a fine powder under liquid N_2_, lysed for 15 min on ice in a five-fold volume of RIPA solution, and centrifuged at ∼10000 **g** for 15 min at 4°C. Supernatants were recovered after high-speed centrifugation and subject to protein concentration quantification via BCA Protein Assay (Pierce, Rockland, IL, USA). Protein lysates were then combined with × 4 Laemmli Sample buffer (final concentration 50 mM Tris-HCl pH 6.8, 100 mM dithiothreitol, 10% glycerol, 0.1% bromophenol blue, 2% SDS), boiled for 10 min, fractionated through 12% Tris-glycine gels (Invitrogen) under reducing conditions, transferred onto Immobilon-P membranes (Millipore, Bedford, MA, USA), and blocked in 1 × PBS containing 5% milk and either 0.3% Tween-20 for c-Myc detection or 0.05% Tween-20 for Bax detection. For Western analysis, blots were incubated for 1 h at room temperature with anti-c-Myc (C-19) or anti-Bax (N-20) antibodies from Santa Cruz Biotechnology (Santa Cruz, CA, USA), washed repeatedly, and incubated in horseradish peroxidase (HRP)-conjugated secondary antibodies (NA934 from Amersham, Buckinghamshire, UK or 611-1302 from Rockland, Gilbertsville, PA, USA for c-Myc; SC-2004 from Santa Cruz). Protein visualisation was achieved using the ECL Western Blotting Reagent Kit (Amersham) and Hyperfilm-ECL photographic film (Amersham).

### Apoptosis and cell proliferation assays

Formalin-fixed, paraffin-embedded mammary tumours were sectioned by microtomy and subsequently utilised to assess the presence and extent of apoptosis in the tumours and surrounding mammary tissues. The terminal deoxynucleotidyl transferase (TdT)-mediated dUTP nick end-labeling (TUNEL) method was used to evaluate apoptosis in these sections (ApopTag Peroxidase *In situ* Apoptosis Detection Kit, Serologicals, Norcross, GA, USA). Briefly, sections were cleared in xylene, rehydrated through an ethanol series, treated with Autozyme Digestion reagent (Biomeda, Foster City, CA, USA), and quenched in 0.15% H_2_O_2_ (Sigma). The TdT reaction was allowed to proceed at room temperature, under humidified conditions, for 40 min and a peroxidase-conjugated anti-digoxigenin antibody was used to assess digoxigenin-dUTP incorporation. A diaminobenzidine/urea chromogen substrate system (Sigma) was used to visualise the TUNEL labeling reaction. Sections were counterstained in 0.5% methyl green dye (Trevigen, Gaithersburg, MD, USA), washed in 100% butanol, and mounted using DPX mountant (Electron Microscopy Sciences). Histological assessment of apoptosis, leading to the calculation of apoptotic index values, was conducted by counting the number of TUNEL-positive apoptotic cells out of >1000 cells in contiguous high-powered (× 40) fields.

Formalin-fixed, paraffin-embedded mammary tumours were sectioned by microtomy, stained with haematoxylin, and utilised to assess the presence and extent of cell proliferation in the tumours and surrounding mammary tissues. Histological assessment of cell proliferation, leading to the calculation of mitotic index values, was conducted by counting the number of mitotic figures out of >1000 cells in contiguous high-powered (× 40) fields.

### Statistical analyses

To evaluate the significance of differences in tumour apoptosis and proliferation between genotypes, all data were subjected to analysis of variance (ANOVA) and Scheffe *post hoc* testing. A Kaplan–Meier curve was generated for the tumour incidence data for the parous study mice and tumour incidence differences between genotypes were assessed using a generalised Wilcoxon test. Analysis of variance testing was utilised to evaluate the significance of tumour multiplicity differences between genotypes.

## RESULTS

### Loss of allelic *bax* alters mammary tumour multiplicity in parous MMTV-c-*myc* transgenic mice

**Figure 1:**
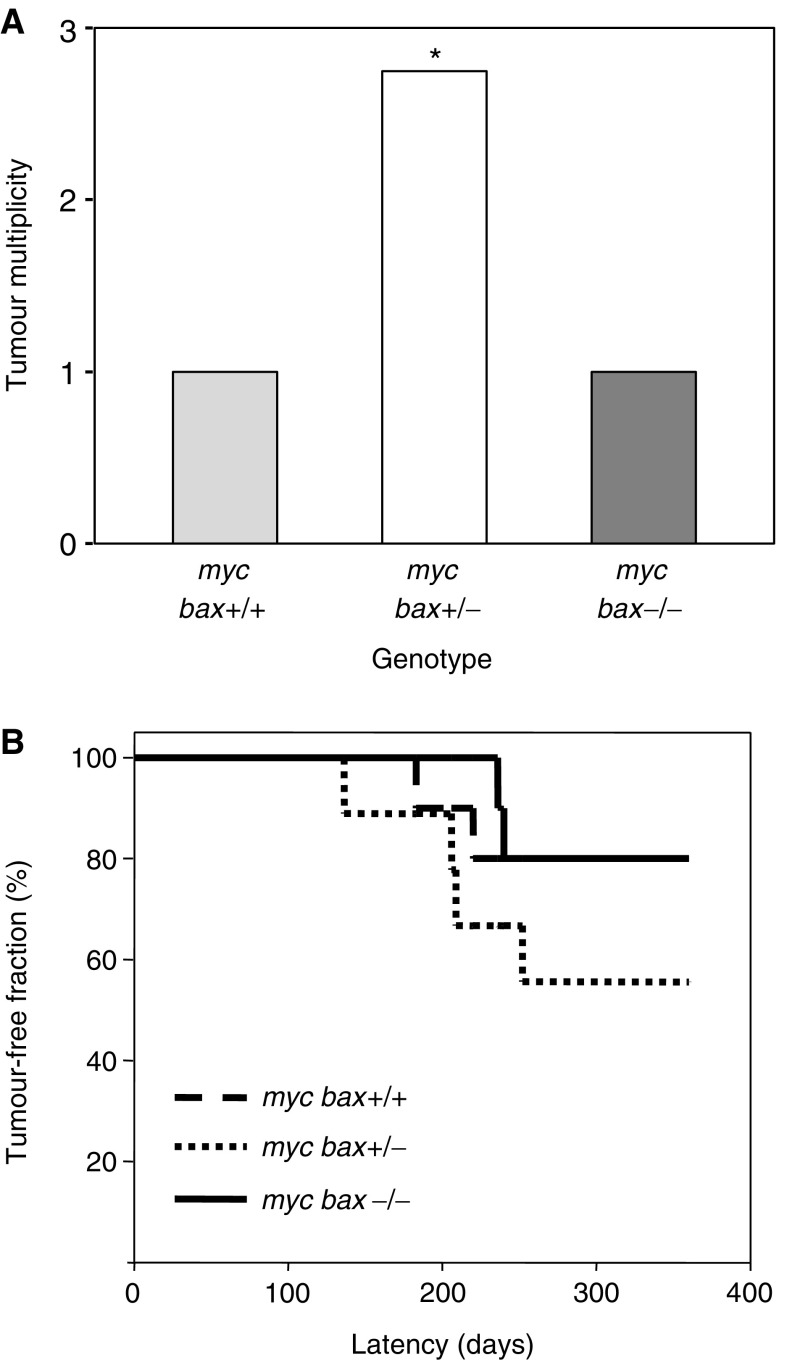
Loss of *bax* influences mammary tumour multiplicity but not mammary tumour incidence in parous MMTV-c-*myc* transgenic/*bax*-knockout mice. (**A**) Mammary tumour multiplicity was increased in parous MMTV-c-*myc*/*bax*-hemizygous transgenic mice (^*^P=0.04 by ANOVA). (**B**) Kaplan–Meier analysis by genotype demonstrates a nonsignificant trend toward decreased tumour-free incidence with *bax*-hemizygosity in parous MMTV-c-*myc* transgenic/*bax*-knockout mice (*P*=0.39 by generalised Wilcoxon testing).

In order to assess the influence of loss of allelic *bax* on c-Myc-induced apoptosis and tumorigenesis in the mammary gland, 10—week-old female study mice (*myc bax*+/+, *n*=10; *myc bax*+/−, *n*=9; *myc bax*−/−, *n*=10) were housed with male mice, bred repetitively, and followed bi-weekly for evidence of mammary tumour development. Parous study mice were euthanised when mammary tumour burden approached 10% of animal body mass or when mice reached 1 year of age (in accordance with US and UKCCR guidelines, Workman *et al*, 1998). Loss of one wild-type *bax* allele in parous c-*myc* transgenic mice elevated mammary tumour multiplicity (2.75 tumours/mouse *vs* 1 tumour/mouse; *P* −0.04 by ANOVA) as compared to parous c-*myc* transgenic mice in which *bax* was intact or completely eliminated (Figure 1A

). A nonsignificant trend towards increased mammary tumour incidence was found for parous MMTV-c-*myc* transgenic/*bax*-hemizygous mice (44.4 *vs* 20 and 20%; *P*=0.39 by Wilcoxon) as compared to parous c-*myc* transgenic mice in which *bax* was intact or completely eliminated (Figure 1B). Mammary tumour latency and parity at time of mammary tumour development were not altered by loss of allelic *bax* in parous MMTV-c-*myc* transgenic mice (data not shown).

Mammary gland whole-mount and haematoxylin and eosin-stained tissue sections were examined for evidence of mammary histopathology. Assessment of mammary gland whole-mounts demonstrated that hyperplastic alveolar nodular changes were present only in mammary glands from tumour-bearing, c-*myc* transgene-positive study mice and were not qualitatively different with loss of allelic *bax* (data not shown). Microscopic histopathological assessment of the sections indicated that the mammary tumours that developed in the parous MMTV-c-myc study mice were cribiform glandular adenocarcinomas as previously described for MMTV-c-*myc* mammary tumours (Cardiff *et al*, 2000). Furthermore, loss of allelic *bax*, in parous MMTV-c-*myc* study mice, did not alter the histopathological character of these mammary tumours nor of the peri-tumorous dysplastic mammary lesions (data not shown).

### c-Myc and Bax expression in mammary tumours and tissue from parous MMTV-c-*myc* transgenic mice

**Figure 2:**
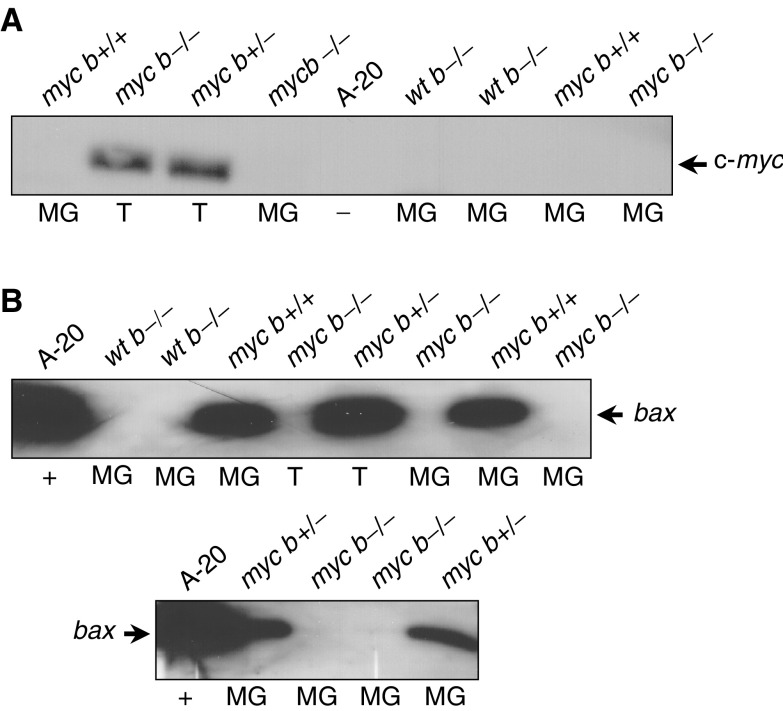
Western blot analysis of transgenic c-Myc protein expression and Bax protein expression in mammary tumours and mammary gland tissues from parous MMTV-c-*myc* transgenic/*bax*-knockout mice. Protein lysates were prepared from mammary tumours (T lanes), mammary gland tissues (MG lanes), and from the A-20 murine lymphoblast cell line (Bax protein positive control). (**A**) Western blot analysis demonstrates expression of c-Myc protein only in mammary tumour lysates from c-*myc* transgene-positive parous study mice. (**B**) Western blot analysis demonstrates that expression of Bax protein is lost in tumour and mammary gland tissue lysates from *bax*-nullizygous parous study mice, that expression of Bax protein is reduced in mammary gland tissue lysates from *bax*-hemizygous parous study mice as compared with *bax*-intact mice, and confirms that *bax* loss of heterozygosity is not evident in mammary tumours from parous MMTV-c-*myc* transgenic/*bax*-hemizygous study mice (Western blots were run simultaneously under identical conditions with mammary gland tissue lysates equally loaded for the amount of protein).

The incidence of mammary tumours in MMTV-c-*myc* transgenic mice, on the FVB inbred genetic background, has been reported to be approximately 50% for virgin female mice and approaching 100% for multiparous female mice (Stewart *et al*, 1984; Amundadottir *et al*, 1995). The overall mammary tumour incidence for multistrain (C57BL/6 × FVB; 3 : 1), multiparous, c-*myc* transgene-positive study mice were 27.6%, considerably lower than previously reported. Western blot analysis was utilised to determine the expression status of the MMTV-c-*myc* transgene in the mammary tumours and mammary gland tissue from parous study mice (c-*myc* transgene-negative mice were included as an assay negative control). c-Myc protein expression was only detectable in lysates from mammary adenocarcinomas and not in lysates from nontumorous mammary gland tissue from c-*myc* transgene-positive mice (Figure 2A

and data not shown).

Western blot analysis confirmed that Bax protein expression was absent in mammary tumour and mammary gland tissue lysates from parous study mice nullizygous for *bax* (Figure 2B). Western blot analysis also confirmed that Bax protein expression was reduced in mammary gland tissue lysates (equally loaded for protein and run simultaneously under identical conditions) prepared from *bax*-hemizygous parous study mice as compared to *bax*-intact parous study mice (Figure 2B). Loss of heterozygosity (LOH) for *bax* was not found to be a part of mammary tumour development and progression in the C3(1)/SV40-*Tag* transgenic/*bax*-hemizygous murine tumour model (Shibata *et al*, 1999) nor in malignancies arising in *arf*-nullizygous/*bax*-hemizygous mice (Eischen *et al*, 2002). *Bax* LOH was not found in mammary tumour development in parous MMTV-c-*myc* transgenic/*bax*-hemizygous mice (Figure 2B and data not shown).

### Loss of allelic *bax* significantly diminishes mammary tumour apoptosis in parous MMTV-c-*myc* transgenic mice

**Figure 3:**
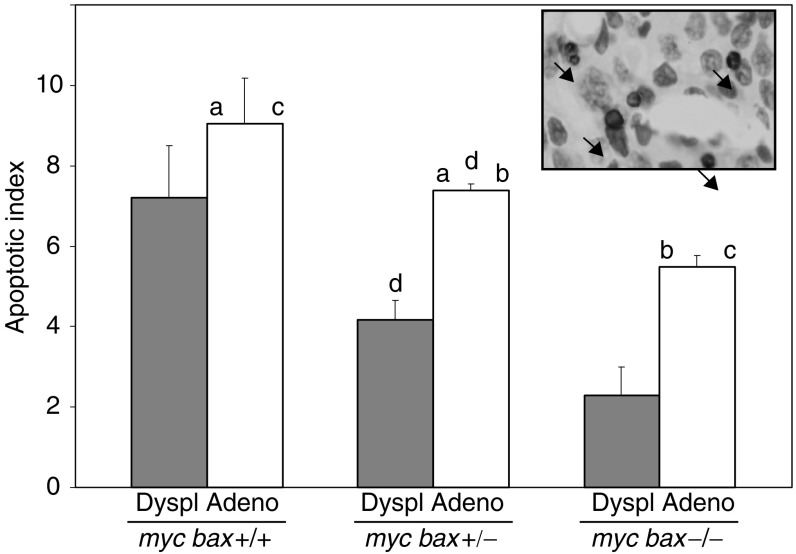
Apoptosis is significantly decreased in mammary adenocarcinomas from parous MMTV-c-*myc* transgenic/*bax*-knockout mice with loss of each wild-type allele of *bax*. A trend toward decreased apoptosis, with loss of allelic *bax*, is seen for mammary dysplastic lesions. Data are presented as mean apoptotic index±s.e.m. and significance comparisons were conducted by ANOVA and Scheffe *post hoc* testing: a (*P*=0.001), b (*P*=0.033), c (*P*=0.017), and d (*P*=0.001). (*Inset*) Histological image of TUNEL-stained mammary section with black arrowheads indicating representative TUNEL-positive apoptotic cells.

Apoptotic indices were generated for both mammary adenocarcinomas and peri-tumorous dysplastic lesions by counting TUNEL-positive cell on mammary tumour and tissue sections. As shown in Figure 3

, apoptosis was significantly decreased in mammary adenocarcinomas with loss of allelic *bax* in parous MMTV-c-*myc* study mice (9.05±1.12 for *myc bax*+/+ *vs* 7.38±0.17 for *myc bax*+/− *vs* 5.48±0.28 for *myc bax*−/−); furthermore, a trend toward diminished apoptosis was seen in mammary dysplastic lesions with loss of allelic *bax* in parous MMTV-c-*myc* study mice (7.20±1.30 for *myc bax*+/+ *vs* 4.17±0.50 for *myc bax*+/− *vs* 2.29±0.71 for *myc bax*−/−). In mammary adenocarcinomas, the levels of apoptosis were significantly different for each of the three evaluated genotypes (*P*=0.001 for *myc bax*+/+ *vs myc bax*+/−; *P*=0.033 for *myc bax*+/− *vs myc bax*−/−; and *P*=0.017 for *myc bax*+/+ *vs myc bax*−/−) as well as between the adenocarcinomas and dysplastic mammary lesions in tumour-bearing, parous MMTV-c-*myc*/*bax*-hemizygous study mice (*P*=0.001).

### Loss of allelic bax does not alter cellular proliferation in mammary tumours from parous MMTV-c-*myc* transgenic mice

**Figure 4:**
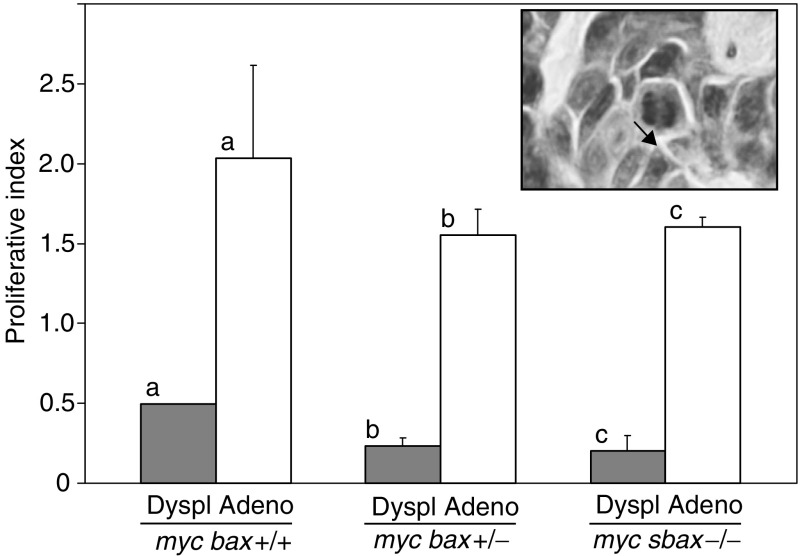
Cellular proliferation is not significantly altered, with loss of allelic *bax*, in mammary adenocarcinomas and mammary dysplastic lesions from parous MMTV-c-*myc* transgenic/*bax*-knockout mice. Cellular proliferation is significantly increased in mammary adenocarcinomas as compared to mammary dysplastic lesions within each genotype. Data are presented as mean proliferative index±s.e.m. and significance comparisons were conducted by ANOVA and Scheffe *post hoc* testing: a (*P*=0.048), b (*P*=0.024), and c (*P*=0.0001). (*Inset*) Histological image of H+E-stained mammary section with black arrowhead indicating a representative mitotic figure.

Proliferative indices were generated for both mammary adenocarcinomas and peritumorous dysplastic lesions by counting mitotic figures on mammary tumour and tissue sections. As shown in Figure 4

, proliferation was not significantly altered in either mammary adenocarcinomas or dysplastic mammary lesions with loss of allelic *bax* in parous MMTV-c-*myc* study mice. Within each genotype, there was significantly more cellular proliferation in mammary adenocarcinomas as compared with peritumorous dysplastic mammary lesions (2.03±0.58 *vs* 0.49±0.005 for *myc bax*+/+, *P*=0.048; 1.55±0.17 *vs* 0.23±0.05 for *myc bax*+/−, *P*=0.024; and 1.60±0.06 *vs* 0.20±0.09 for *myc bax*−/−, *P*=0.0001).

## DISCUSSION

The exogenous expression of murine c-*myc* using the MMTV-LTR promoter system has previously been demonstrated to induce mammary tumorigenesis; furthermore, these mammary tumours are characterised by a significantly elevated apoptotic index (Stewart *et al*, 1984; Amundadottir *et al*, 1996). Results from the MMTV-c-*myc*/MT-*tgfα* bitransgenic mammary tumour model suggest that a diminution of *in vivo* apoptosis can accentuate c-Myc-induced mammary tumour formation (Amundadottir *et al*, 1995). The Bax protein is known to be a key mitochondrial regulator of apoptosis and has been shown to be a transcriptional target of c-Myc (Mitchell *et al*, 2000). In this latter capacity, Bax may be responsible, in part, for apoptosis resulting from deregulated c-*myc* expression and its loss in human breast tumours may eliminate c-Myc's potential tumour suppressive role. In this study, *bax*-knockout and MMTV-c-*myc* transgenic mice were mated to generate a mammary-relevant model in which the influence of the loss of *bax* on c-Myc-induced apoptosis and tumorigenesis could be investigated. Our results clearly demonstrate that loss of *bax* is directly and significantly correlated with a reduction in apoptosis in mammary adenocarcinomas. Furthermore, haploid loss of *bax*, in the context of MMTV-c-*myc* expression, results in an elevation in mammary tumour multiplicity without influencing mammary tumour latency or histopathology. However, in contradistinction to prior findings for c-Myc-mediated lymphomagenesis (Eischen *et al*, 2001a), complete loss of *bax* did not promote c-Myc-induced mammary tumorigenesis, suggesting that some amount of Bax expression is required for mammary tumorigenesis.

Previous studies have reported the incidence of mammary tumours in single strain, multiparous MMTV-c-*myc* mice as approaching 100%; however, no studies of MMTV-c-*myc* transgenic or *myc*-containing bitransgenic mice have reported mammary tumour multiplicity findings (Stewart *et al*, 1984; Jamerson *et al*, 2000). In our study of multiparous, multistrain MMTV-c-*myc* transgenic mice possessing both wild-type *bax* alleles, the incidence of mammary tumours was 20% and the multiplicity was one tumour per mouse. This reduced mammary tumour incidence identified in our studies for c-*myc* transgenic mice may reflect the tumour suppressive influences of a mixed-strain background in our mouse model or may represent functional changes in the genetic control of the c-*myc* transgene itself. Significantly, other studies have concluded that alteration of or mixing of inbred genetic backgrounds can significantly influence transgene-induced mammary tumorigenesis (Griep *et al*, 1998; Lifsted *et al*, 1998; Rowse *et al*, 1998; Le Voyer *et al*, 2000; Rose-Hellekant *et al*, 2002). The C57BL/6 XFVB cross utilised in the current study has been previously studied in this respect, implicating C57BL/6 as bearing an unknown mammary tumour penetrance-modifying influence (Rose-Hellekant *et al*, 2002). Although the c-Myc penetrance-modifier in our study remains unknown, methylation of the MMTV-LTR promotional element, silencing of linked transgene expression, and abrogation of transgene-dependent tumorigenesis has been described previously (Mangues *et al*, 1995; Betzl *et al*, 1996; Zhou *et al*, 2001). Additionally, MMTV-LTR methylation patterns are heterogeneous among offspring from the same litter and promoter demethylation appears to be required for transgene-driven tumorigenesis. The reduced mammary tumour incidence in our study animals may result from methylation of the MMTV-c-*myc* transgene and concomitant reduction or elimination of c-Myc protein expression. Analysis of c-Myc protein expression in mammary gland and tumour lysates confirms that c-Myc expression is limited to the mammary adenocarcinomas and is below the sensitivity of this assay in the nontumorous mammary glands from c-*myc* transgene-positive and transgene-negative mice. These data suggest that functional changes in the genetic or epigenetic control of c-*myc* transgene expression may be responsible for the diminished penetrance of the mammary tumour phenotype in our studies.

Evaluation of mammary gland whole mounts from tumour-bearing and nontumour-bearing study mice revealed a definite correlation between the presence of multiple hyperplastic alveolar nodules and the presence of c-*myc* transgene expression and mammary adenocarcinomas. The absence of mammary gland hyperplastic changes, as assessed by whole mount, in animals that do not express the c-*myc* transgene lends credence to the idea that the transforming influence of c-*myc* transgene expression is required for hyperplastic, dysplastic, and neoplastic changes in the mammary gland in this model. In tumour-bearing mice, the presence of transgene-induced glandular changes at the whole mount level is confirmed by the identification of mammary gland dysplastic changes at the microscopic histopathological level. As expected, and independent of the status of *bax* in our study mice, a cribiform mammary adenocarcinoma phenotype was identified. Evaluation of the mammary gland pathology of genetically engineered mice has shown that the c-*myc* transgene-induced adenocarcinomas are characterised by a cribiform phenotype that is dominantly expressed when the c-*myc* transgene is co-expressed with other transgenes (Cardiff *et al*, 2000). Therefore, our findings revealed the transforming role of the c-*myc* transgene in our tumour model and have suggested that expression of the cribiform tumour phenotype is not abrogated by elimination of the *bax* tumour suppressor gene.

As anticipated for an apoptosis-regulatory gene, loss of wild-type *bax* alleles did not significantly alter proliferation, in both mammary adenocarcinomas and dysplastic mammary tissue, in tumour-bearing MMTV-c-*myc* mice. Our results did demonstrate a significant increase in cellular proliferation between dysplastic mammary tissues and mammary adenocarcinomas, as might be expected in the progression of mammary lesions. As expected with the loss of the proapoptotic *bax* gene, a trend toward diminished apoptosis in dysplastic mammary tissue and a significant diminution in apoptosis in mammary adenocarcinomas was identified in tumour-bearing MMTV-c-*myc* mice with loss of one and both wild-type alleles of *bax*. Our studies also demonstrate that haploid loss of *bax* is associated with an increase in mammary tumour multiplicity in multiparous MMTV-c-*myc* study mice; intriguingly however, complete loss of the wild-type *bax* alleles results in a mammary tumour multiplicity, but not incidence identical to that for mice with intact *bax*. These results, of *bax* loss influencing mammary gland apoptosis and mammary tumour multiplicity, but not incidence, are similar, but not identical, to those seen for the C(3)1/SV40-*Tag*/*bax*-knockout mice (Shibata *et al*, 1999). Characterisation of *bax*-hemizygous and *bax*-nullizygous mice expressing the C(3)1-*Tag* transgene resolved that selectively in hemizygous *bax* animals, apoptosis was significantly reduced in preneoplastic mammary lesions with subsequent enhancement of tumour number (Shibata *et al*, 1999). No such enhancement in SV40-dependent mammary tumorigenesis was observed in *bax-*nullizygous mice. The reductions in c-Myc-mediated mammary tumour multiplicity and incidence seen in our *bax*-nullizygous mice are similar to the findings reported from this previous study and may reflect mammary gland hypoplasia resulting from loss of both wild-type alleles of *bax* (Shibata *et al*, 1999). It is worth considering that Bax may have a stage-specific role in suppressing c-Myc-mediated mammary tumorigenesis (apoptosis suppression with *bax* loss is differentially stage-specific with respect to the inducer of apoptosis, Myc or TAg). Notably, as indicated earlier in the introduction, Bax loss strongly enhances c-Myc-dependent lymphomagenesis (Eischen *et al*, 2001a). It should be noted that c-Myc-dependent lymphomagenesis is also strongly enhanced in the absence of one or both p53 alleleles, in contrast to mammary tumorigenesis. (Elson *et al*, 1995, McCormack *et al*, 1998). Since c-Myc-dependent mammary tumours seldom contain mutated p53, in striking contrast to lymphomas, future studies could productively address the interactions between p53 and Bax in distinguishing differential, c-Myc effects on tumorigenesis in these two tissue types.

In conclusion, haploid loss of *bax* in multiparous MMTV-c-*myc* transgenic mice is associated with a significantly decreased mammary tumour apoptotic index. Loss of *bax* is not associated with alterations in mammary tumour proliferation. Our results indicate that *bax* is involved in the regulation of apoptosis in tumours of the murine mammary gland and may be, at best, a weakly negative modulator of c-Myc-mediated mammary tumorigenesis. Complete loss of *bax*, associated with the most significant suppression of apoptosis in mammary tumours in our model, is clearly not associated with suppression of mammary tumorigenesis, as compared with loss of one wild-type allele of *bax*. This finding suggests that Bax may be required for mammary tumour development at some stage, and that the contribution of other apoptotic pathways may be important to mammary tumorigenesis in our model. These results are the first published that demonstrate that haploid loss of *bax*, as seen in many human breast cancers, significantly reduces mammary tumour apoptosis provoked by a human breast cancer-relevant proto-oncogene, c-*myc*. Our results clearly show that in contrast to the role of Bax as a proapoptotic tumour suppressor in c-Myc-induced lymphomogenesis (Eischen *et al*, 2001a), in c-Myc-dependent mammary tumorigenesis Bax is proapoptotic, but lacking in significant mammary tumour suppressive activity.

## References

[bib1] Amundadottir LT, Johnson MD, Merlino G, Smith GH, Dickson RB (1995) Synergistic interaction of transforming growth factor alpha and c-myc in mouse mammary and salivary gland tumorigenesis. Cell Growth Diff 6: 737–748 7669729

[bib2] Amundadottir LT, Nass SJ, Berchem GJ, Johnson MD, Dickson RB (1996) Cooperation of TGF alpha and c-Myc in mouse mammary tumorigenesis: coordinated stimulation of growth and suppression of apoptosis. Oncogene 13: 757–765 8761297

[bib3] Antonsson B, Conti F, Ciavetti A, Montessuit S, Lewis S, Martinou I, Bernasconi L, Bernard A, Mermod JJ, Mazzei G, Maundrell K, Gambale F, Sadoul R, Martinou JC (1997) Inhibition of Bax channel-forming activity by Bcl-2. Science 277: 370–372 9219694 10.1126/science.277.5324.370

[bib4] Antonsson B, Montessuit S, Lauper S, Eskes R, Martinou JC (2000) Bax oligomerization is required for channel-forming activity in liposomes and to trigger cytochrome *c* release from mitochondria. Biochem J 345: 271–278 10620504 PMC1220756

[bib5] Bargou RC, Daniel PT, Mapara MY, Bommert K, Wagener C, Kallinich B, Royer HD, Dörken B (1995) Expression of the Bcl-2 gene family in normal and malignant breast tissue: low Bax-alpha expression in tumor cells correlates with resistance towards apoptosis. Int J Cancer 60: 854–859 7896458 10.1002/ijc.2910600622

[bib6] Bargou RC, Wagener C, Bommert K, Mapara MY, Daniel PT, Arnold W, Dietel M, Guski H, Feller A, Royer HD, Dörken B (1996) Overexpression of the death-promoting gene Bax-alpha which is downregulated in breast cancer restores sensitivity to different apoptotic stimuli and reduces tumor growth in SCID mice. J Clin Invest 97: 2651–2659 8647960 10.1172/JCI118715 PMC507353

[bib7] Bates SE, Valverius EM, Ennis BW, Bronzert DA, Sheridan JP, Stampfer MR, Mendelsohn J, Lippman ME, Dickson RB (1990) Expression of the transforming growth factor-alpha/epidermal growth factor receptor pathway in normal human breast epithelial cells. Endocrinology 126: 596–607 2294006 10.1210/endo-126-1-596

[bib8] Betzl G, Brem G, Weidle UH (1996) Epigenetic modification of transgenes under the control of the mouse mammary tumor virus LTR: tissue-dependent influence on transcription of the transgenes. Biol Chem 377: 711–719 8960372 10.1515/bchm3.1996.377.11.711

[bib9] Cardiff RD, Anver MR, Gusterson BA, Hennighausen L, Jensen RA, Merino MJ, Rehm S, Russo J, Tavassoli FA, Wakefield LM, Ward JM, Green JE (2000) The mammary pathology of genetically engineered mice: the Consensus report and recommendations from the Annapolis meeting. Oncogene 19: 968–988 10713680 10.1038/sj.onc.1203277

[bib10] D'Cruz CM, Gunther DJ, Boxer RB, Hartman JL, Sintasath L, Moody SE, Cox JD, Ha SI, Belka GK, Golant A, Cardiff RD, Chodosh LA (2001) c-Myc induces mammary tumorigenesis by means of a preferred pathway involving spontaneous Kras2 mutations. Nat Med 7: 235–239 11175856 10.1038/84691

[bib11] Dang CV (1999) c-Myc target genes involved in cell growth, apoptosis and metabolism. Mol Cell Biol 19: 1–11 9858526 10.1128/mcb.19.1.1 PMC83860

[bib12] Deming SL, Nass SJ, Dickson RB, Trock BJ (2000) c-Myc amplification in breast cancer: a meta-analysis of its occurrence and prognostic relevance. Br J Cancer 83: 1688–1695 11104567 10.1054/bjoc.2000.1522 PMC2363455

[bib13] Desagher S, Osen-Sand A, Nichols A, Eskes R, Montessuit S, Lauper S, Maundrell K, Antonsson B, Martinou JC (1999) Bid-induced conformational change of Bax is responsible for mitochondrial cytochrome *c* release during apoptosis. J Cell Biol 144: 891–901 10085289 10.1083/jcb.144.5.891 PMC2148190

[bib14] Eischen CM, Rehg JE, Korsmeyer SJ, Cleveland JL (2002) Loss of Bax alters tumor spectrum and tumor numbers in ARF-deficient mice. Cancer Res 62: 2184–2191 11929842

[bib15] Eischen CM, Roussel MF, Korsmeyer SJ, Cleveland JL (2001a) Bax loss impairs c-Myc-induced apoptosis and circumvents the selection of p53 mutations during Myc-mediated lymphomagenesis. Mol Cell Biol 22: 7653–7662 10.1128/MCB.21.22.7653-7662.2001 PMC99936 11604501

[bib16] Eischen CM, Woo D, Roussel MF, Cleveland JL (2001b) Apoptosis triggered by Myc-induced suppression of Bcl-xL or Bcl-2 is bypassed during lymphomagenesis. Mol Cell Biol 21: 5063–5070 11438662 10.1128/MCB.21.15.5063-5070.2001 PMC87232

[bib17] Elson A, Deng C, Campos-Torres J, Donchower LA, Leder P (1995) The MMTV/c-myc transgene and p53 null alleles collaborate to induce T-cell lymphomas, but not mammary carcinomas in transgenic mice. Oncogene 11: 181–190 7624126

[bib18] Evan GI, Littlewood TD (1993) The role of c-Myc in cell growth. Curr Opin Genet Dev 3: 44–49 8453273 10.1016/s0959-437x(05)80339-9

[bib19] Evan GI, Wyllie AH, Gilbert CS, Littlewood TD, Land H, Brooks M, Waters CM, Penn LZ, Hancock DC (1992) Induction of apoptosis in fibroblasts by c-Myc protein. Cell 69: 119–128 1555236 10.1016/0092-8674(92)90123-t

[bib20] Felsher DW, Bishop JM (1999a) Transient excess of Myc activity can elicit genomic instability and tumorigenesis. Proc Natl Acad Sci USA 96: 3940–3944 10097142 10.1073/pnas.96.7.3940 PMC22399

[bib21] Felsher DW, Bishop JM (1999b) Reversible tumorigenesis by Myc in hematopoietic lineages. Mol Cell 4: 199–207 10488335 10.1016/s1097-2765(00)80367-6

[bib22] Feuerhake F, Sigg W, Höfter EA, Dimpfl T, Welsch U (2000) Immunohistochemical analysis of Bcl-2 and Bax expression in relation to cell turnover and epithelial differentiation markers in the non-lactating human mammary gland epithelium. Cell Tissue Res 299: 47–58 10654069 10.1007/s004419900127

[bib23] Griep AE, Krawcek J, Lee D, Liem A, Albert DM, Carabeo R, Drinkwater N, McCall M, Sattler C, Lasudry JG, Lambert PF (1998) Multiple genetic loci modify risk for retinoblastoma in transgenic mice. Invest Ophthalmol Vis Sci 39: 2723–2732 9856783

[bib24] Harrington EA, Bennett MR, Fanidi A, Evan GI (1994) c-Myc-induced apoptosis in fibroblasts is inhibited by specific cytokines. EMBO J 13: 3286–3295 8045259 10.1002/j.1460-2075.1994.tb06630.x PMC395225

[bib25] Jäger R, Herzer U, Schenkel J, Weiher H (1997) Overexpression of Bcl-2 inhibits alveolar cell apoptosis during involution and accelerates c-*myc*-induced tumorigenesis of the mammary gland in transgenic mice. Oncogene 15: 1787–1795 9362445 10.1038/sj.onc.1201353

[bib26] Jamerson MH, Johnson MD, Dickson RB (2000) Dual regulation of proliferation and apoptosis: c-*myc* in bitransgenic murine mammary tumor models. Oncogene 19: 1065–1071 10713691 10.1038/sj.onc.1203268

[bib27] Jhappan C, Stahle C, Harkins RN, Fausto N, Smith GH, Merlino GT (1990) TGF-alpha overexpression in transgenic mice induces liver neoplasia and abnormal development of the mammary gland and pancreas. Cell 61: 1137–1146 2350785 10.1016/0092-8674(90)90076-q

[bib28] Juin P, Hueber AO, Littlewood T, Evan GI (1999) c-Myc-induced sensitization to apoptosis is mediated through cytochrome c release. Genes Dev 13: 1367–1381 10364155 10.1101/gad.13.11.1367 PMC316765

[bib29] Juin P, Hunt A, Littlewood T, Griffiths B, Swigart LB, Korsmeyer SJ, Evan GI (2002) c-Myc functionally cooperates with Bax to induce apoptosis. Mol Cell Biol 22: 6158–6169 12167710 10.1128/MCB.22.17.6158-6169.2002 PMC133996

[bib30] Jurgensmeier JM, Xie Z, Deveraux Q, Ellerby L, Bredesen D, Reed JC (1998) Bax directly induces release of cytochrome *c* from isolated mitochondria. Proc Natl Acad Sci USA 95: 4997–5002 9560217 10.1073/pnas.95.9.4997 PMC20202

[bib31] Kapranos N, Karaiosifidi H, Valavanis C, Kouri E, Vasilaros S (1997) Prognostic significance of apoptosis related proteins Bcl-2 and Bax in node-negative breast cancer patients. Anticancer Res 17: 2499–2506 9252670

[bib32] Knudson CM, Tung KSK, Tourtellotte WG, Brown GAJ, Korsmeyer SJ (1995) Bax-deficient mice with lymphoid hyperplasia and male germ cell death. Science 270: 96–99 7569956 10.1126/science.270.5233.96

[bib33] Krajewski S, Blomqvist C, Franssila K, Krajewska M, Wasenius VM, Niskanen E, Nordling S, Reed JC (1995) Bcl-2 family proteins and the regulation of programmed cell death in leukemia and lymphoma. Cancer Res 55: 4471–4478 7671262

[bib34] Krajewski S, Krajewska M, Shabaik A, Miyashita T, Wang HG, Reed JC (1994) Immunohistochemical determination of the *in vivo* distribution of Bax, a dominant inhibitor of Bcl-2. Am J Pathol 145: 1323–1336 7992838 PMC1887502

[bib35] Le Voyer T, Lu Z, Babb J, Lifsted T, Williams M, Hunter KW (2000) An epistatic interaction controls the latency of a transgene-induced mammary tumor. Mamm Genome 11: 883–889 11003704 10.1007/s003350010163

[bib36] Leder A, Pattengale PK, Kuo A, Stewart TA, Leder P (1986) Consequences of widespread deregulation of the c-Myc gene in transgenic mice: multiple neoplasms and normal development. Cell 45: 485–495 3011271 10.1016/0092-8674(86)90280-1

[bib37] Li M, Hu J, Heermeier K, Hennighausen L, Furth PA (1996) Apoptosis and remodeling of mammary gland tissue during involution proceeds through p53-independent pathways. Cell Growth Diff 7: 13–20 8788029

[bib38] Lifsted T, Le Voyer T, Williams M, Muller W, Klein-Szanto A, Buetow KH, Hunter KW (1998) Dentification of inbred mouse strains harboring genetic modifiers of mammary tumor age of onset and metastatic progression. Int J Cancer 77: 640–644 9679770 10.1002/(sici)1097-0215(19980812)77:4<640::aid-ijc26>3.0.co;2-8

[bib39] Mangues R, Schwartz S, Seidman I, Pellicer A (1995) Promoter demethylation in MMTV/N-*ras* transgenic mice required for transgene expression and tumorigenesis. Mol Carcinogen 14: 94–102 10.1002/mc.2940140205 7576104

[bib40] Matsui Y, Halter SA, Holt JT, Hogan BLM, Coffey RJ (1990) Development of mammary hyperplasia and neoplasia in MMTV-TGF alpha transgenic mice. Cell 61: 1147–1155 2161707 10.1016/0092-8674(90)90077-r

[bib41] McCormack SJ, Weaver Z, Deming S, Natarajan G, Torri J, Johnson MD, Liyanage M, Ried T, Dickson RB (1998) Myc/p53 interactions in transgenic mouse mammary development, tumorigenesis, and chromosal instability. Oncogene 16: 2755–2766 9652742 10.1038/sj.onc.1201804

[bib42] Mitchell KO, Ricci MS, Miyashita T, Dicker DT, Jin Z, Reed JC, El-Deiry WS (2000) Bax is a transcriptional target and mediator of c-Myc-induced apoptosis. Cancer Res 60: 6318–6325 11103792

[bib43] Murphy KM, Streips UN, Lock RB (1999) Bax membrane insertion during Fas(CD95)-induced apoptosis precedes cytochrome *c* release and is inhibited by Bcl-2. Oncogene 18: 5991–5999 10557088 10.1038/sj.onc.1203001

[bib44] Nass SJ, Dickson RB (1997) Defining a role for c-Myc in breast tumorigenesis. Breast Cancer Res Treat 44: 1–22 9164674 10.1023/a:1005858611585

[bib45] Nouraini S, Six E, Matsuyama S, Krajewski S, Reed JC (2000) The putative pore-forming domain of Bax regulates mitochondrial localization and interaction with Bcl-xL. Mol Cell Biol 20: 1604–1615 10669738 10.1128/mcb.20.5.1604-1615.2000 PMC85344

[bib46] Oltvai ZN, Milliman CL, Korsmeyer SJ (1993) Bcl-2 heterodimerizes *in vivo* with a conserved homolog, Bax, that accelerates programmed cell death. Cell 74: 609–619 8358790 10.1016/0092-8674(93)90509-o

[bib47] Packham G, Cleveland JL (1995) c-Myc and apoptosis. Biochim Biophys Acta 1242: 11–28 7626652 10.1016/0304-419x(94)00015-t

[bib48] Prendergast GC (1999) Mechanisms of apoptosis by c-Myc. Oncogene 18: 2967–2987 10378693 10.1038/sj.onc.1202727

[bib49] Rose-Hellekant TA, Gilchrist K, Sandgren EP (2002) Strain background alters mammary gland lesion phenotype in transforming growth factor-a transgenic mice. Am J Pathol 161: 1439–1447 12368216 10.1016/s0002-9440(10)64419-7 PMC1867309

[bib50] Rowse GJ, Ritland SR, Gendler SJ (1998) Genetic modulation of neu proto-oncogene-induced mammary tumorigenesis. Cancer Res 58: 2675–2679 9635596

[bib51] Sakakura C, Sweeney EA, Shirahama T, Igarashi Y, Hakomori S, Nakatani H, Tsujimoto H, Imanishi T, Ohgaki M, Ohyama T, Yamazaki J, Hagiwara A, Yamaguchi T, Sawai K, Takahashi T (1996) Overexpression of Bax sensitizes human breast cancer MCF-7 cells to radiation-induced apoptosis. Int J Cancer 67: 101–105 8690508 10.1002/(SICI)1097-0215(19960703)67:1<101::AID-IJC17>3.0.CO;2-H

[bib52] Sandgren EP, Luetteke NC, Palmiter RD, Brinster RL, Lee DC (1990) Overexpression of TGF alpha in transgenic mice: induction of epithelial hyperplasia, pancreatic metaplasia, and carcinoma of the breast. Cell 61: 1121–1135 1693546 10.1016/0092-8674(90)90075-p

[bib53] Sandgren EP, Schroeder JA, Qui TH, Palmiter RD, Brinster RL, Lee DC (1995) Inhibition of mammary gland involution associated with transforming growth factor *α* but not c-*myc*-induced tumorigenesis in transgenic mice. Cancer Res 55: 3915–3927 7641211

[bib54] Santoni-Rugiu E, Jensen MR, Thorgeirsson SS (1998) Disruption of the pRb/E2F pathway and inhibition of apoptosis are major oncogenic events in liver constitutively expressing c-Myc and transforming growth factor alpha. Cancer Res 58: 123–134 9426068

[bib55] Schorr K, Li M, Bar-Peled U, Lewis A, Heredia A, Lewis B, Knudson CM, Korsmeyer SJ, Jäger R, Weiher H, Furth PA (1999a) Gain of Bcl-2 is more potent than Bax loss in regulating mammary epithelial cell survival *in vivo*. Cancer Res 59: 2541–2545 10363969

[bib56] Schorr K, Li M, Krajewski S, Reed JC, Furth PA (1999b) Bcl-2 gene family and related proteins in mammary gland involution and breast cancer. J Mamm Gland Biol Neoplasia 4: 153–164 10.1023/a:1018773123899 10426394

[bib57] Shibata MA, Liu ML, Knudson MC, Shibata E, Yoshidome K, Bandey T, Korsmeyer SJ, Green JE (1999) Haploid loss of Bax leads to accelerated mammary tumor development in C3(1)/SV40-Tag transgenic mice: reduction in protective apoptotic response at the preneoplastic stage. EMBO J 18: 2692–2701 10329616 10.1093/emboj/18.10.2692 PMC1171351

[bib58] Shilkaitis A, Graves J, Mehta RR, Hu L, You M, Lubet R, Steele V, Kelloff G, Christov K (2000) Bcl-2 and bax are differentially expressed in hyperplastic, premalignant, and malignant lesions of mammary carcinogenesis. Cell Growth Diff 11: 437–445 10965848

[bib59] Sjöström J, Krajewski S, Franssila K, Niskanen E, Wasenius VM, Nordling S, Reed JC, Blomqvist C (1998) A multivariate analysis of tumour biological factors predicting response to cytotoxic treatment in advanced breast cancer. Br J Cancer 78: 812–815 9743306 10.1038/bjc.1998.584 PMC2062970

[bib60] Snedeker SM, Brown CF, DiAugustine RP (1991) Expression and functional properties of transforming growth factor alpha and epidermal growth factor during mouse mammary gland ductal morphogenesis. Proc Natl Acad Sci USA 88: 276–280 1986376 10.1073/pnas.88.1.276 PMC50793

[bib61] Soucie EL, Annis MG, Sedivy J, Filmus J, Leber B, Andrews DW, Penn LZ (2001) Myc potentiates apoptosis by stimulating Bax activity at the mitochondria. Mol Cell Biol 21: 4725–4736 11416148 10.1128/MCB.21.14.4725-4736.2001 PMC87151

[bib62] Stewart TA, Pattengale PK, Leder P (1984) Spontaneous mammary adenocarcinomas in transgenic mice that carry and express MTV/myc fusion genes. Cell 38: 627–637 6488314 10.1016/0092-8674(84)90257-5

[bib63] Strange R, Li F, Saurer S, Burkhardt A, Friis RR (1992) Apoptotic cell death and tissue remodeling during mouse mammary gland involution. Development 115: 49–58 1638991 10.1242/dev.115.1.49

[bib64] Telang NT, Osborne MP, Sweterlitsch LA, Narayanan R (1990) Neoplastic transformation of mouse mammary epithelial cells by deregulated Myc expression. Cell Regul 1: 863–872 2088530 10.1091/mbc.1.11.863 PMC362853

[bib65] Valverius EM, Ciardiello F, Heldin NE, Blondel B, Merlo G, Smith GH, Stampfer MR, Lippman ME, Dickson RB, Salomon DS (1990) Stromal influences on transformation of human mammary epithelial cells overexpressing c-Myc and SV40T. J Cell Physiol 145: 207–216 2174061 10.1002/jcp.1041450204

[bib66] Vennstrom B, Sheiness D, Zabielski J, Bishop JM (1982) Isolation and characterization of c-Myc, a cellular homolog of the oncogene (v-Myc) of avian myelocytomatosis virus strain 29. J Virol 42: 773–779 6284994 10.1128/jvi.42.3.773-779.1982 PMC256910

[bib67] Weaver ZA, McCormack SJ, Liyanage M, du Manoir S, Coleman A, Schröck E, Dickson RB, Ried T (1999) A recurring pattern of chromosomal aberrations in mammary gland tumors of MMTV-c-myc transgenic mice. Genes Chromosome Cancer 25: 251–260 10379871

[bib68] Wei MC, Zong WX, Cheng EHY, Lindsten T, Panousakopoulou V, Ross AJ, Roth KA, MacGregor GR, Thompson CB, Korsmeyer SJ (2001) Proapoptotic BAX and BAK: a requisite gateway to mitochondrial dysfunction and death. Science 292: 727–730 11326099 10.1126/science.1059108 PMC3049805

[bib69] Workman P, Twentyman P, Balkwill F, Balmain A, Chaplin D, Double J, Embleton J, Newell D, Raymond R, Stables J, Stephens T, Wallace J (1998) United Kingdom Co-ordinating Committee on Cancer Research (UKCCCR) Guidelines for the Welfare of Animals in Experimental Neoplasia (Second Edition). Br J Cancer 77: 1–10 10.1038/bjc.1998.1 PMC2151254 9459138

[bib70] Zhou H, Chen W, Qin X, Lee K, Liu L, Markowitz SD, Gerson SL (2001) MMTV promoter hypomethylation is linked to spontaneous and MNU-associated c-*neu* expression and mammary carcinogenesis in MMTV-c-*neu* transgenic mice. Oncogene 20: 6009–6017 11593408 10.1038/sj.onc.1204830

[bib71] Zindy F, Eischen CM, Randle DH, Kamijo T, Cleveland JL, Sherr CJ, Roussel MF (1998) Myc signaling via the ARF tumor suppressor regulates p53-dependent apoptosis and immortalization. Genes Dev 12: 2424–2433 9694806 10.1101/gad.12.15.2424 PMC317045

